# Incorporating cancer risk information into general practice: a qualitative study using focus groups with health professionals

**DOI:** 10.3399/bjgp17X689401

**Published:** 2017-02-14

**Authors:** Juliet A Usher-Smith, Barbora Silarova, Alison Ward, Jane Youell, Kenneth R Muir, Jackie Campbell, Joanne Warcaba

**Affiliations:** Primary Care Unit, University of Cambridge, Cambridge.; MRC Epidemiology Unit, University of Cambridge, Cambridge.; Institute of Health and Wellbeing, University of Northampton, Northampton.; Institute of Health and Wellbeing, University of Northampton, Northampton.; Institute of Population Health, University of Manchester, Manchester.; Institute of Health and Wellbeing, University of Northampton, Northampton.; Moulton Surgery, Moulton.

**Keywords:** behaviour change, cancer, prevention, primary care, risk assessment

## Abstract

**Background:**

It is estimated that approximately 40% of all cases of cancer are attributable to lifestyle factors. Providing people with personalised information about their future risk of cancer may help promote behaviour change.

**Aim:**

To explore the views of health professionals on incorporating personalised cancer risk information, based on lifestyle factors, into general practice.

**Design and setting:**

Qualitative study using data from six focus groups with a total of 24 general practice health professionals from the NHS Nene Clinical Commissioning Group in England.

**Method:**

The focus groups were guided by a schedule covering current provision of lifestyle advice relating to cancer and views on incorporating personalised cancer risk information. Data were audiotaped, transcribed verbatim, and then analysed using thematic analysis.

**Results:**

Providing lifestyle advice was viewed as a core activity within general practice but the influence of lifestyle on cancer risk was rarely discussed. The word ‘cancer’ was seen as a potentially powerful motivator for lifestyle change but the fact that it could generate health anxiety was also recognised. Most focus group participants felt that a numerical risk estimate was more likely to influence behaviour than generic advice. All felt that general practice should provide this information, but there was a clear need for additional resources for it to be offered widely.

**Conclusion:**

Study participants were in support of providing personalised cancer risk information in general practice. The findings highlight a number of potential benefits and challenges that will inform the future development of interventions in general practice to promote behaviour change for cancer prevention.

## INTRODUCTION

It is estimated that approximately 40% of all cases of cancer are attributable to lifestyle factors such as smoking, alcohol consumption, diet, weight, and physical activity, and nearly 600 000 cancer cases in the UK could have been avoided in the past 5 years if people had healthier lifestyles.^1^ Prevention strategies are likely to require a combination of approaches that target the underlying determinants of cancer at both population and individual levels. With more than 300 million consultations taking place each year,^2^ general practice provides an ideal platform from which to deliver individual-level interventions. It is already the largest site of delivery for the NHS Health Check programme and brief interventions based in general practice have been shown to reduce smoking^3^^,^^4^ and alcohol consumption,^5^ as well as promoting physical activity 6^,^^7^ and weight loss.^8^^,^^9^ With the exception of smoking cessation, however, almost all of these interventions focus on cardiovascular disease (CVD); there is little discussion about cancer.

A number of risk tools are now available that predict an individual’s future risk of cancer. These include the Harvard Risk Tool,^10^^,^^11^ QCancer,^12^^,^^13^ and large numbers of disease-specific tools (for example, the Gail risk model for breast cancer).^14^ Each tool uses risk factors, such as age, sex, family history of cancer, and medical history, alongside lifestyle factors to estimate an individual’s risk of developing cancer over a defined time period, often 10 years.

A number of theories of health behaviour change suggest that risk perception may be an important factor associated with preventive behaviours.^15^^,^^16^ Being able to estimate and communicate individual risk, and demonstrate the impact of lifestyle change on future risk of cancer, therefore, has the potential to motivate behaviour change. A recent systematic review of cancer risk assessment tools in primary care suggests their use has potentially beneficial effects on the accuracy of patient risk perception and knowledge, intentions to have cancer screening, and changes in diet and physical activity, without causing an increase in cancer-specific anxiety.^17^

Unlike similar risk tools for other conditions (such as QRisk2,^18^ Predict CVD,^19^ the Framingham risk score for CVD,^20^ the FRAX fracture risk assessment tool,^21^ and QFracture),^22^ these tools for cancer are not integrated into primary care computer systems or routinely used in practice. The aim of this study, therefore, was to explore health professionals’ views on the potential for incorporating into general practice personalised cancer risk information based on lifestyle factors to improve understanding of cancer prevention and promote behaviour change.

**How this fits in**

Approximately 40% of all cases of cancer are attributable to lifestyle factors. General practice interventions have been shown to be effective in lifestyle modification but have not routinely targeted cancer. In this study, primary care health professionals supported the provision of personalised cancer risk information and numerical risk estimates in general practice. They identified a number of potential benefits and challenges, and the findings can inform the future development of interventions to promote behaviour change for cancer prevention in general practice.

## METHOD

### Design

A qualitative study was carried out, using data collected from focus groups with health professionals.

### Participants and recruitment

GPs, GP registrars, practice nurses, student nurses, and clinical commissioners were recruited from practices within the NHS Nene Clinical Commissioning Group (CCG). Nene CCG plans and commissions healthcare services in the East Midlands in England on behalf of a population of approximately 650 000 and includes 69 GP surgeries. Four recruitment strategies were used:

- GP surgeries across Nene CCG were approached and invited to take part in the study. If a surgery expressed an interest in taking part, all health professionals at that practice were individually emailed a letter of invitation and the study information leaflet;
- all GPs within a local professional development group were individually emailed and invited to take part;
- letters of invitation and the study information leaflet were emailed to all current GP registrars and student nurses through the Northampton GP training scheme coordinator and the University of Northampton nursing course portal; and
- details of the study and the invitation to take part were circulated with the weekly newsletter to all Nene CCG members.

Recruitment took place between November 2015 and February 2016, ending only when data saturation was achieved.

### Data collection

Each focus group was led by two researchers and guided by a schedule (available from authors on request) to explore:

- participants’ views on presenting personalised information about the risk of developing common cancers in general; and
- future implementation.

**Figure 1.**
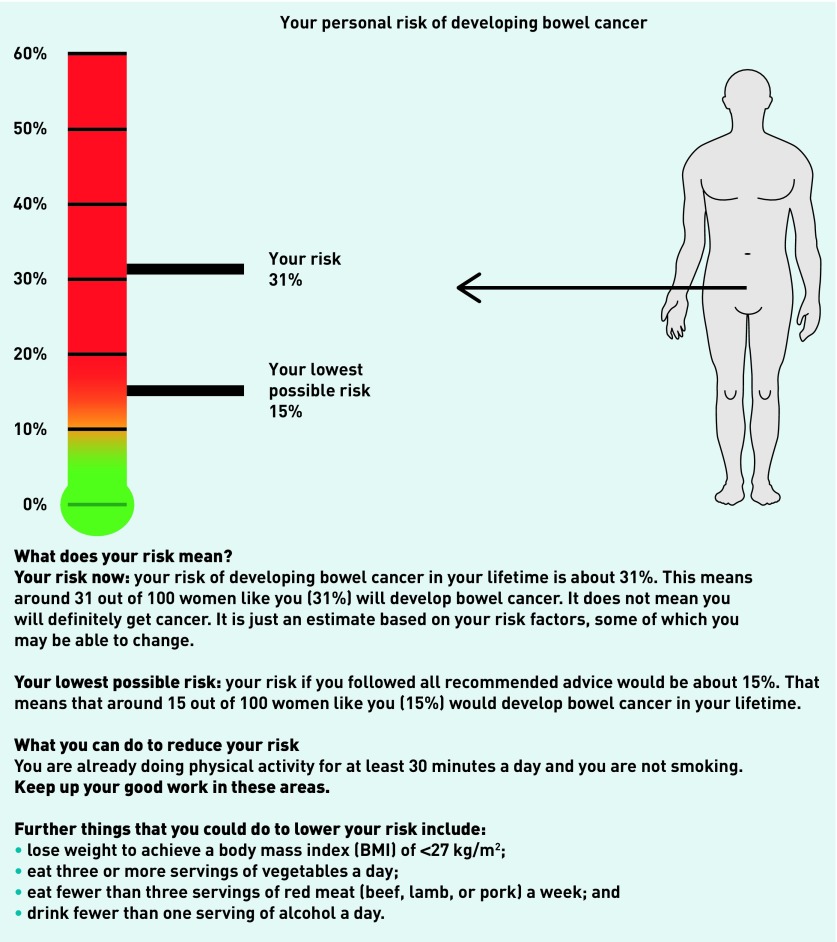
***Diagrammatic example of a personalised risk estimate providing the 10-year absolute risk of colon cancer for a hypothetical patient shown to focus group participants.***

For those focus groups involving GPs and primary care teams, this included whether they felt that the provision of such information is within the scope of primary care and, if so, how this would be integrated. For the focus groups with clinical commissioners, the discussion focused mainly on funding and service provision. In all groups, participants were first asked how people in their practice currently get information about their future risk of disease and lifestyle advice in general, and about cancer specifically. They were then shown an example of a personalised risk estimate (Figure 1); this illustrated the 10-year risk of colon cancer for a hypothetical 65-year-old female now and if she made lifestyle changes.

### Analysis

Focus groups were audiotaped and transcribed verbatim, then analysed using thematic analysis with the aid of NVivo software (version 10). After reading and re-reading the transcripts, two researchers developed a coding frame.

Once coding was completed by the same two researchers and any discrepancies resolved following discussion with a third researcher, a written summary of the data was created for each code using the ‘one sheet of paper’ method^23^ — this involves every section of data relevant to that code from all the focus groups being noted. This allowed identification of the key themes within the data and any outlying views. These themes were then explored in depth, looking for any patterns across different professional groups, sex, and years of experience.

The data were analysed as they were collected in order to assess when data saturation was reached. This initial analysis was performed by four researchers (a UK GP, a health psychologist, and two non-clinical researchers experienced in qualitative health research), with the final interpretation agreed by all seven researchers.

## RESULTS

A total of 24 general practice health professionals took part across six focus groups, with three to five participants per group. Three groups were held in GP practices with GPs, nurses, and student nurses, two with GP registrars and primary care nurses within the University of Northampton, and one with clinical commissioners at Nene CCG’s offices. Participant characteristics are given in Table 1. Five key themes were identified from the data:

- current lack of focus on the role of lifestyle factors in cancer;
- the power of the word ‘cancer’;
- presentation of personalised cancer risk;
- facilitators and barriers to incorporating cancer risk information into general practice; and
- alternative settings.

**Table 1.** Participants’ characteristics (*n* = 24)

**Characteristic**	***n***
**Sex**	
Male	10
Female	14

**Profession**	
GP	11
GP clinical commissioner	3
Nurse	7
Clinical commissioner	3

**Experience**	
In training	8
<5 years	4
5–10 years	5
>10 years	7

Apart from the anticipated different perspective provided by the clinical commissioners around funding and service provision, there were no differences between the views of GPs and nurses, males and females, or those with different levels of experience.

For context, job titles and years of experience of participants are given after quotations.

### Lack of focus on the role of lifestyle factors in cancer

Providing health promotion and lifestyle advice was viewed as a core activity in general practice. Most of this work was carried out by nurses via the NHS Health Check programme or in dedicated appointments initiated by patients or through GP referrals. It was unusual for GPs to have specific time for health promotion and they tended to fit it in during other appointments. Most of the advice given during those consultations was focused on CVD; although GPs recognised that many of the risk factors overlap with those for cancer, cancer was only raised opportunistically — and mostly in relation to smoking and lung cancer:

*‘I don’t think* … *I specifically address cancer risk in those consultations, it’s more opportunistic for me so like* … *if you do a smear* [test]*, that kind of cancer risk crops up. But just general cancer risk, I think if I’m talking about risk with a patient it’s generally cardiovascular risk. I don’t think at the moment I really mention cancer unless it’s linked to smoking.’*

(GP 5, focus group 4, 5–10 years)

Those involved in commissioning were also not aware of any current services targeting cancer prevention:

‘*I don’t think we do many or if any, you know, sort of cancer risk assessments or planning for cancer in that sense*.’

(GP commissioner 2, focus group 5, 5–10 years)

In addition, although all participants were familiar with, and regularly used, risk assessment tools for CVD, none was aware of any similar tools for cancer.

### The power of the word ‘cancer’

Several participants in each group mentioned the power of the word ‘cancer’, and there was a general view that people may make more lifestyle changes in response to risk of cancer than to risk of CVD because they perceive cancer to be more serious, or are more afraid of it:

‘I think this might be quite a positive in terms of changing their lifestyle because I think someone is more likely to change their lifestyle when presented with a cancer risk than when presented with a heart attack or a stroke risk, because I think heart attack and a stroke most people recover, you know, but cancer I think people think it’s a lot more serious.’

(GP 4, focus group 4, >10 years)

*‘I think certainly some people* [would make lifestyle changes]*, purely out of the fact that some people are so fearful of the word ‘cancer’, as soon as you mention it they probably want to do something. If you talk about the risk of getting dementia or the risk of getting heart disease in the future it doesn’t seem to strike them as much, but as soon as you mention the C word, they probably act a lot more, I think.’*

(GP trainee 2, focus group 3, in training)

This was particularly thought to be the case for younger people, for whom cancer was felt to be more relevant than CVD:

*‘I think cancer’s more, especially in the young* … *it’s something that they can relate to because they may know of someone with cancer, whereas cardiovascular disease I think they see as something* [that] *happens to older people and not something they need to worry about right now.’*

(GP 2, focus group 1, <5 years)

There was acknowledgement, however, of the challenges of trying to get people to change their behaviour; one participant likened it to ‘*bang*[ing] *our heads against brick walls*’ (GP trainee 2, Focus group 3, in training). The potential for discussions about cancer risk to be demotivating if patients perceived the risk to be high, and therefore unchangeable, and the possibility of generating health anxiety and reinforcing anxiety in those already concerned about their health were also raised across the focus groups:

‘… you want to make sure that the information you’re giving people is not only understandable but that it doesn’t completely alter them in a way that’s unhelpful, because you don’t want to induce health anxiety because that’s, you know, that doesn’t help us or them mostly.’

(GP trainee 3, focus group 3, in training)

*‘It* [the risk estimate] *may be quite high and a bit demotivating if you think “well no matter what I do, you know, I’m going to get cancer”, which might be what some people take away from this.’*

(GP 4, focus group 4, >10 years)

Several individuals, however, discussed how cardiovascular risk assessment and faecal occult blood testing had generated anxiety when they were first introduced, but people ‘*just expect it now*’ (student nurse 1, focus group 1, in training). Normalising cancer risk by making it part of routine care was, therefore, seen as an important way to reduce the potential for generating anxiety:

‘So if it was more of a routine type, you know, check, but for the younger age group or whatever, then that would normalise it, wouldn’t it? So perhaps the anxiety wouldn’t be as great.’

(Nurse 1, focus group 1, >10 years)

*‘But it’s all about what people expect at the moment and what we’re doing so maybe not now, but maybe in 5 years, everyone will expect a letter about their cancer risk when they turn 30, like they expect their smear* [test] *letter. And if they know that’s coming, then it’s more acceptable isn’t it?’*

(GP 2, focus group 1, <5 years)

### Presentation of personalised cancer risk

Many participants felt providing a personalised cancer risk would be more likely to influence behaviour than generic information:

‘I think a generic cancer message is less likely to hit home, or it’s less likely to influence an individual … I think people need to see the relevance to them … so personalising it makes it a bit more likely that they will absorb the information and do something about it.’

(GP 7, focus group 2, >10 years)

Almost all also thought the risk should be presented as a numerical estimate. They felt that providing numbers makes the assessment *‘a more powerful tool*’ (GP trainee 1, focus group 3, in training), with patients feeling *‘it’s more personalised or applicable to them*’ (GP 2, focus group 1, <5 years).

Several participants felt numbers made it easier for patients to understand, and referred specifically to their experience of discussions around the risks of hormone replacement therapy (HRT) to illustrate this:

*‘I found it really useful prescribing HRT using the table in the* BNF [*British National Formulary*], *which has actual numbers for risk of breast cancer and thromboembolism and, if you give the patient actual numbers they seem to respond to that better than just saying “you have an increased risk” … I think it’s easier for patients to understand.’*

(GP 5, focus group 4, 5–10 years)

There was much discussion in all groups about the relative advantages and disadvantages of presenting the risk of individual cancers, compared with presenting a combined risk for the most common cancers or those for which lifestyle factors have the biggest impact. All participants felt that there was a place for risk scores for individual cancers to be used with patients presenting with concerns about one or more cancers or wanting specific details. However, the general consensus was that a combined risk — perhaps with details of the top cancers contributing to that risk — might be better for general health promotion:

‘I think patients might want overall, but it’s a bit of a fluffy concept … if you just give them their overall cancer risk it’s hard for them to think about specifics. But if we’re really targeting it for health promotion, if we just did the overall cancer risk then actually it’s just about getting them to think about it, and that would get them to think about it …’

(GP 5, focus group 4, 5–10 years)

Some participants went further and felt that, as the lifestyle advice was similar to that for cancer and CVD prevention, separating them was potentially overcomplicating the message and more confusing — an overall health promotion message might be more effective.

‘*… you’re at 31% risk of cancer … and by the way you’ve got a 15% chance of stroke, cardiovascular as well … so I think it needs to be seen as sort of a broader health.*’

(Student nurse 2, focus group 6, in training)

### Facilitators and barriers

All groups felt that the NHS should be providing information about cancer risk, with the majority believing there was value in providing it face to face and that general practice was the most appropriate setting:

*‘… we’re best placed to do it out of everyone, the best place to have that little bit* [of] *extra time, the face-to-face contact, and the opportunity to do it.’*

(GP 5, focus group 4, 5–10 years)

There was recognition among all participants that much of the advice they already provide is relevant to cancer, and so adding advice about cancer into practice would be relatively straightforward:

‘I think it’s just the overlap with cardiovascular disease, we’re doing a lot of it already so we probably do it quite well anyway.’

(GP 3, focus group 4, 5–10 years)

*‘You wouldn’t be asking the nurses or the healthcare assistants to ask* [for] *any more information than they already do — they already ask their weight and their diet and their family history and their alcohol — and it would be a springboard for the rest of their consult, so it would be kind of a useful, you know, it would cover a few bases.’*

(GP trainee 3, focus group 3, in training)

As with current lifestyle services, it was felt that most advice would come from nurses and healthcare assistants, with patients at high risk referred to GPs for a further discussion. The challenge was finding the right time and accessing the right group of people:

‘You have to have appropriate risk provided at appropriate times and that’s the key isn’t it really, so people have to be willing to hear the message.’

(GP commissioner 1, focus group 5, 5–10 years)

‘*People who worry about their health obsessively are the ones we see more and they’re already worried about having cancer and probably already doing most things right … whereas you want to get the message out to the people who are not in the doctor’s surgery.*’

(GP 3, focus group 4, 5–10 years)

Across the groups, four main options to provide information about cancer risk within general practice were identified:

- opportunistically;
- within NHS Health Checks;
- within existing cancer screening programmes; or
- within new-patient health appointments.

Most participants felt happy to discuss cancer risk opportunistically, but there was a general consensus that, although providing the information to older patients might be helpful, the focus should be on targeting younger people in their 20s and 30s for whom any lifestyle changes would have the greatest impact. In addition, focus group members felt that offering cancer risk estimates to people who attend general practice is likely to identify mostly older people and those who are already health conscious. A small number of GPs were also unsure how comfortable they would feel introducing the subject of cancer if it had not already come up in some way during the consultation:

‘I’d be happy using this tool — there’s a lot of positive things about it — but I don’t know how happy I’d be using it just cold, in a sort of health promotion-type setting without giving the patient some kind of inkling that it might be coming.’

(GP 4, focus group 4, >10 years)

Adding cancer risk information to NHS Health Checks or sending information out with letters about national screening programmes was felt to make sense and to fit nicely:

*‘Yeah,* [fitting it into NHS Health Checks] *would make perfect sense because they’ve got all the information anyway because they’re doing all the risk, they’re doing all the lifestyle stuff anyway aren’t they?’*

(GP 6, focus group 4, 5–10 years)

However, it was recognised that the NHS Health Check programme was targeted at older people and that screening programmes, such as cervical screening, focus on women more than men. As a result, appointments for new patients were felt to be the least biased:

‘I think women do engage with health concerns more than men. That’s why if you had an avenue to collect people in a way that’s not centred around this, but you could pick it up during that contact, so like the health questionnaires or a new patient, you know — everybody’s got to have a GP so you could incorporate it in there quite nicely and that way you wouldn’t get a population bias.’

(GP trainee 3, focus group 3, In training)

Whichever route was chosen, however, there was a clear need for additional resources if it was going to be offered widely. Participants mentioned the need for additional funding and time:

‘So it would have to be … funded as an enhanced service if that was something that was going to be delivered in primary care in its own right, unless it was just an optional thing you say like many other things we know, you know, we have training on.’

(GP 6, focus group 4, 5–10 years)

‘I think anything would have to be funded wouldn’t it? Because we’re kind of really stretched as it is.’

(Nurse 2, focus group 1, >10 years)

*‘… if someone came in and said “Oh I’m worried about my cancer risk, what can you tell me?” and then you thought “Oh I’ve seen that risk score, I’ll get that up and do it” but that would be a very rare scenario in primary care that someone would actually come in specifically asking about that one issue. They’ll generally come in about something else* … *I just think realistically it wouldn’t get done unless it was sort of dedicated time.’*

(GP 6, Focus group 4, 5–10 years)

Referral pathways were thought to be needed to support lifestyle change:

‘And the other thing would depend on what avenues we had open to us to follow up … I mean I see quite a lot of patients now who are worried about family history and we end up referring to clinical genetics with the family history questionnaires, and that’s fine because you know you’ve got something to offer them … whereas with this we haven’t got a pathway.’

(GP 6, focus group 4, 5–10 years)

Participants also saw the importance of this being integrated into the computer system:

‘… if you want to get that out to every practice and every GP, then it needs to be integrated.’

(Clinical commissioner 1, focus group 5, <5 years)

‘… the computer system needs to be able to extract everything that it wants without having to type in extra information at the time, and you need to be able to click on a button and it just instantly spits out the whole thing ready for you, you know. You don’t want to be having to go and re-enter, sort of, how many bits of fruit they ate in the last week before it’ll produce its thing.’

(GP 8, focus group 2, >10 years)

The point that screening resources might also need to improve was also raised:

‘I think you may have increased demand again from patients in terms of saying, “Well my risk is this high, I want this test and I want this screening, you know, I want to be screened.” So as long as that’s accommodated for as well, and that’s thought about.”

(GP trainee 1, focus group 3, in training)

It was also recognised that training would be needed. One nurse (Nurse 2, focus group 1, >10 years) commented that *‘… everybody needs training’*, whereas a GP noted:

‘Anyone could give you advice if they were trained to, trained in the risk tool and understood it.’

(GP 4, focus group 4, >10 years)

Additional barriers mentioned by those involved in commissioning services included the continued reorganisation of services over the last 10 years, movement of public health out of health care into the local authorities, and the current instability of funding:

‘Public health was part of the health care, not part of the local authority, so you could actually agree pathways and can work together to do that, but now they’re within local authority and the politics of local authority is completely different, you know, it’s a different agenda.’

(Clinical commissioner 3, focus group 5, <5 years)

‘The continued reorganisation that’s gone on over the last 10 or so years has meant that smoking cessation has migrated from one community of providers to another … if it’s constantly changing so that even the GP isn’t sure where the service is being provided, then that’s probably not so helpful.’

(GP commissioner 1, focus group 5, 5–10 years)

### Alternative settings

In addition to providing cancer risk information in general practice, all the groups felt it was important to increase the accessibility of the information, so people did not have to visit their GP, and to make use of technology to engage younger people. Suggestions included:

- providing face-to-face consultations in pharmacies, supermarkets, and schools;
- allowing individuals to assess their own risk via websites, mobile applications, or ‘cancer booths’, potentially supported by videos to explain how to interpret the results; and
- offering risk assessment by post.

As one participant said:

‘You could get a booth couldn’t you, a booth, like a photo booth and you plug in all your things and then it goes “your cancer risk is 30%”.’

(GP trainee 3, focus group 3, in training)

The potential for increasing anxiety was again raised in these discussions, however, with recognition that there was a balance between accessibility and interpretation:

‘… the only problem with making it too accessible is the interpretation. If a patient sees that and goes “Oh my God, I’m going to die” … actually you’re there to interpret the information in the surgery.’

(GP 3, focus group 4, 5–10 years)

## DISCUSSION

### Summary

This study showed that, among the UK health professionals who took part, there is support for providing personalised cancer risk information in general practice. The findings highlighted a number of potential benefits and challenges, which could inform the future development of interventions to promote behaviour change for cancer prevention in general practice. These include:

- the power of the word ‘cancer’ to motivate lifestyle change, but recognition of its potential to also generate health anxiety;
- the preference for risk to be presented as a numerical estimate for both an overall cancer risk and individual cancers;
- the challenge of finding the right time and place to provide the risk information, and accessing those most likely to benefit; and
- the need for additional resources, including funding, time, referral pathways, integration within the electronic health record, and training.

### Strengths and limitations

To the authors’ knowledge this is the first study detailing the views of health professionals on incorporating personalised cancer risk information into general practice in the UK. A diverse sample of health professionals was recruited in terms of sex, profession, and level of experience. The use of semi-structured, qualitative data collection allowed their views on key areas to be explored while areas they felt were important could be raised.

There are some limitations that should be noted, however. The sample size was small and all participants were recruited from one region in England, which limits the generalisability of the findings. The individuals and practices that took part were also those that chose to give up their time to participate, so it is possible they may be more interested in the subject and more supportive of using personalised cancer risk information than health professionals in the wider community.

### Comparison with existing literature

The findings from this study are consistent with those from similar studies undertaken in the US,^24^^–^27 Australia,^28^ and France.^29^ As in those studies, there was not only support for providing cancer risk information in practice, but also recognition that trying to get people to change their behaviour is difficult,^26^^,^^27^ and there is a need for additional funding,^28^ time,^24^^–^28 integration into the current electronic health record,^29^ and training^24^^,^^29^ for implementation to be successful.

One of the biggest challenges raised was finding the best time and place for the risk assessment. Participants in the study reported here felt that general practice was the most appropriate setting and that, ideally, the risk should be delivered face to face. This was also the view of breast surgeons and primary care physicians in the study in Australia.^28^ Other studies have, however, demonstrated the feasibility of providing the information via the internet,^30^ as a leaflet,^31^ or incorporated within screening programmes.^32^

The findings that almost all participants in this study thought the risk should be presented as a numerical estimate and that personalised messages were more likely to influence behaviour than messages directed towards the entire population is also consistent with previous reports.^26^^,^^33^

### Implications for research and practice

Much of the lifestyle advice already provided in relation to CVD is relevant to cancer, so adding advice about cancer into practice could be relatively straightforward. However, this study highlighted a number of factors that should be considered when incorporating cancer risk information into general practice. The first is the format and content of the risk information. This study suggests addressing issues by:

- presenting a numerical estimate, with details of how that would be reduced in response to lifestyle changes for a combined cancer risk and the most common individual cancers;
- providing information face to face in general practice; and
- targeting younger people and those most likely to benefit from lifestyle changes, perhaps at new-patient health checks.

Health professionals, however, often have difficulty interpreting and communicating risk scores to patients.^34^^,^^35^ In addition, previous studies on presenting cancer risk to individuals have highlighted the difficulties patients have understanding the concept of risk.^36^^,^^37^ It is also unlikely that one strategy will suit all and so further research is needed with both health professionals and patients to identify the optimal method of information provision.

The second is the need to provide additional resources, particularly time, funding, and referral pathways. At a time when demand for health care is increasing and funding decreasing, these are likely to be the greatest barriers. The ongoing reorganisation of the NHS and movement of public health away from health care into the remit of local authorities will also make introducing new prevention services more challenging. Any future initiatives will require the support not only of GPs and clinical commissioners, but also of public-health and third-sector organisations. Providing risk information via the internet, mobile applications, or written leaflets instead may be more cost-effective but this must be balanced against the risk of generating anxiety.

Finally, before personalised cancer risk information is introduced in any format, there must be some assurance that health professionals will not be causing harm. A recent systematic review of cancer risk assessment tools in primary care suggests their use could have beneficial effects without causing an increase in cancer-specific anxiety;^17^ however, this hypothesis was based on only three trials so further research is needed to quantify the potential benefits and harms of providing cancer risk information in routine general practice.
